# IL13Rα1 protects against rheumatoid arthritis by combating the apoptotic resistance of fibroblast-like synoviocytes

**DOI:** 10.1186/s13075-020-02270-4

**Published:** 2020-08-08

**Authors:** Xiaomei Yang, Qingwei Guo, Tingting Feng, Qiqi Lu, Luna Ge, Jihong Pan, Kehong Bi, Li Qiao, Lei Tian, Tianhua Xie, Chengfang Yao, Guanhua Song, Lin Wang

**Affiliations:** 1 Department of Hematology, Qilu Children’s Hospital of Shandong University, Jinan, China; 2 Shandong Provincial Qianfoshan Hospital, Shandong University, Jinan, China; 3 Department of Pathology, Shandong University Medical School, Jinan, China; 4 Department of Rheumatology and Autoimmunology, The First Affiliated Hospital of Shandong First Medical University, Key Lab for Biotech-Drugs of National Health Commission, Key Lab for Rare & Uncommon Diseases of Shandong Province, Shandong Medicinal Biotechnology Centre, Jinan, 250062 China; 5 School of Medicine and Life Sciences, University of Jinan-Shandong Academy of Medical Sciences, Jinan, China; 6 Department of Joint Surgery, Shandong Provincial Hospital Affiliated to Shandong University, Jinan, China; 7 Department of Rheumatology, Shandong Provincial Hospital Affiliated to Shandong University, Jinan, China; 8 Institute of Basic Medicine, Shandong First Medical University & Shandong Academy of Medical Sciences, Jinan, 250062 China

**Keywords:** Rheumatoid arthritis, ER stress, IL13Rα1, Apoptosis, Synoviocyte

## Abstract

**Background:**

Endoplasmic reticulum (ER) stress is closely related with the pathological progression of rheumatoid arthritis (RA), and fibroblast-like synoviocytes (FLSs) are known as its resistance against ER stress-induced apoptosis. Studies on overcoming such resistance would provide a novel treatment strategy for RA in a clinical setting.

**Methods:**

IL13Rα1 expression was assessed in the synovial tissue by RT-qPCR, immunohistology, and Western blot. Gain or loss of functional analysis was applied to evaluate the biological roles of IL13Rα1 in RA FLSs. Cell viability and apoptosis were assessed by MTS, Western blot, and flow cytometry. The therapeutic effects of IL13Rα1 on the severity of type II collagen-induced arthritis (CIA) in DBA-/1 mouse model were evaluated by scoring synovitis, hyperplasia, cartilage degradation, and bone destruction.

**Results:**

IL13Rα1 expression was selectively downregulated when RA FLSs were stimulated by ER stress inducers. Functionally, IL13Rα1 overexpression could inhibit the viability, but induce the apoptosis of RA FLSs in the presence of ER stress inducers. Mechanistically, IL13Rα1 promotes cell apoptosis via transcriptionally activating trail expression. Besides, IL13Rα1 could interact and stabilize DR5 protein, thus forming a positive loop involving trail and DR5 to render RA FLSs more susceptible to apoptosis. Additionally, intraarticular injection of IL13Rα1 conferred therapeutic effects in CIA models and showed a limited degree of synovial proliferation and joint destruction.

**Conclusions:**

Together, our data establishes a regulatory role for IL13Rα1 to combat the apoptotic resistance of RA FLSs against ER stress. The inhibitory effects of IL13Rα1 on arthritis progression suggest the therapeutic potential in RA.

## Introduction

Endoplasmic reticulum (ER) stress is a common cellular response in rheumatoid arthritis (RA) [1, 2]. Hypoxia, glucose deprivation, reactive oxygen species, and pro-inflammatory cytokines could induce ER stress, thereby provoking the excessive accumulation of unfolded protein in both innate immune cells (e.g., dendritic cells, adaptive immune cells (e.g., T and B cells), and mesenchymal stromal cells (fibroblast-like synoviocytes (FLSs)) in the inflamed joints [3]. Although unfolded protein response (UPR) can alleviate sustained ER stress by inhibiting protein translation, activating chaperones, and enhancing proteasome degradation of misfolded proteins, the persistent ER stress will turn on the proapoptotic arm of UPR via inducing ChoP expression [4]. Several studies have highlighted the importance of ER stress as its effects on cellular apoptosis, proliferation, inflammation, and their potential consequences for synovial hyperplasia [5, 6]. RA FLSs produce large amounts of cytokines and enzymes, and 30% of all newly synthesized, ER-sorted proteins are unfolded [1, 3, 5]. However, unlike other cell types, RA FLSs are resistant to ER stress-induced apoptosis and the mechanistic basis underlying for it remains still unclear [7]. Identifying local factors that are able to combat this resistance is therefore of great importance to understand the pathological characters and also potential intervention for RA.

Numerous studies reported that IL-13could be viewed as a serum biomarker and therapeutic target for RA [8–11]. Besides its anti-inflammatory role, IL-13 can also protect human synovial cells from apoptosis and promote synovial proliferation [5, 12]. IL13Rα1 is required for delivering IL-13 responses of fibroblasts, eosinophils, and dendritic cells to affect the pathological features associated with asthma and lung injury [8]. Upon binding to IL-13, IL13Rα1 forms a heterodimer with IL-4Rα, internalizes into the cytosol and consequently activates cytosolic signal transducer and activator of transcription 6 (STAT6) [9]. Importantly, it has been reported that IL-13 could induce ER stress and promote apoptosis and death in microglia [13]. Furthermore, ER stress can impair IL-13 signaling via repressing IL4Rα [14]. However, the precise roles of its receptors in the cellular response towards ER stress are still unclear in RA. In addition, IL13Rα1 has no intrinsic kinase activity but rather constitutively associates Tyk2 via its proline-rich Box-1 region in the cytoplasm [8]. Intriguingly, the increased pathology of pulmonary injury in bleomycin-treated IL13Rα1^−/−^ mice may be largely independent of IL-13 [12]. Hence, it is important to define the activity and the signaling pathway downstream of IL13Rα1 under ER stress.

Trail has several receptors include the agonistic receptors DR4 and DR5 [15–17], the decoy receptors Trail-R3 (DcR1) and Trail-R4 (DcR2), which antagonize the death signal [18, 19], and osteoprotegerin (OPG) [20]. DR5 (Trail-R2) was highly expressed in the synovial tissue and fibroblasts from RA and specific activation of Trail-DR5 signaling was effective for ameliorating the arthritis phenotype of RA [21]. However, the regulatory mechanism towards Trail-DR5 remains largely unknown in RA FLSs.

In this study, we found that RA FLSs with IL13Rα1 overexpression are more susceptible to apoptosis in response to ER stress. More importantly, the pro-apoptotic feature of IL13Rα1 is also functional even in the absence of its ligands IL-13. At the molecular level, IL13Rα1-mediated activation of STAT6 transcriptionally upregulates trail expression. Meanwhile, IL13Rα1 interacts and stabilizes DR5 protein on the cell surface of RA FLSs. Therefore, IL13Rα1 activates the Trail-DR5 signaling pathway and promotes apoptosis of RA FLSs. We also explore the therapeutic potential of IL13Rα1 and demonstrate that IL13Rα1 delivery into joint ameliorates the severity of collagen-induced arthritis (CIA).

## Materials and methods

### Patients

Human synovial tissues were collected from a total of 26 RA patients fulfilling the 1987 criteria of the American College of Rheumatology [22] and obtained at the time of joint replacement surgery at the Shandong Provincial Hospital and Shandong Qiaofo Mountain Hospital (Jinan, China). Twelve osteoarthritis (OA) patients [23] who underwent total joint replacement. The mean age of the RA patients (12 males and 14 females) was 54.8 ± 10.9 years. The mean disease duration was 62.3 mo (range 9–159 months). The mean ages of the OA patients were 54.7 ± 7.8 years. The majority of RA patients were treated with disease-modifying antirheumatic drugs (DMARDs), including methotrexate, and none were on biologics. Demographic data are provided in Supplementary Table 1. The study protocol was approved by the Institutional Review Board of Shandong Medicinal Biotechnology Center (VCMC08BR067). All patients gave fully informed written consent approved by the institutional ethics committee, and research was performed in accordance with the Declaration of Helsinki.

### Reagents and cell stimulation

RA FLSs were cultured overnight to reach 80% confluence in medium containing 1% fetal cells and subsequently stimulated with the following reagent. Cycloheximide (CHX, 10 μg/mL, 239,763) was purchased from Calbiochem. Thapsigargin (Tg, T9033) and Tunicamycin (Tm, 654,380) were classical inducers for ER stress and purchased from Sigma. Cobalt chloride (CoCl_2_) was bought from Sigma Company. STAT6 inhibitor (100 nM, AS1517499) and IL-13 (213-ILB) were purchased from Selleck and R&D Systems, respectively.

### Cell isolation and culture

FLSs were obtained from the synovial tissues of RA or OA patients as described previously [2] and cells between passages 4 and 7 were used for further study. Cells were cultured in Dulbecco’s modified Eagle’s medium (DMEM) with 10% FBS in a 37 °C 5% CO_2_ incubator (Thermo, USA). To mimic the hypoxic condition, RA FLSs were treated with CoCl_2_ as described before [24].

### Lentiviral transduction

Myc-tagged-wild type human and mouse IL13Rα1 (wt) was constructed and cloned into pcDNA3.1/Zeo(+) (Thermo Fisher Scientific) by Cyagen Biosciences (Guangzhou, China). Lentivirus products were constructed in HEK293T cells by co-transfection with psPAX2, pMD2.G, and lentiviral construct (pLentiCMV/TO Puro DEST) expressing wild type IL13Rα1 (Lv-IL13Rα1) using lipofectamine 2000 (Invitrogen, Carlsbad, CA, USA).

### siRNA transfection and apoptosis

RA FLSs (2 × 10^5^ cells in 100-mm-diameter dishes or 8.5 × 10^4^ cells in 6-well plates) were plated to reach 75% confluence and were transiently transfected with IL13Rα1 or DR5 small interfering RNA (siRNA, Ruibo Biosciences, Guanzhou, China) by Hiperfect transfection reagent (QIAGEN) following the manufacturer’s instructions, and all experiments were performed 24–48 h after transfection. The silencing efficiency siRNAs targeting IL13Rα1 or DR5 were measured by Western blot, and the two showing obviously silencing efficiencies were selected and combined at the equal concentration for following experiments. The sequences of siRNA targeting human IL13Rα1 (siIL13Rα1) and human DR5 (siDR5) were as followings: siIL13Rα1#1: GGAAACTCGTCGTTCAATA; siIL13Rα1#2: GGAGCCAGCTCAAATTGTA; siDR5#1: CAGCCGUAGUCUUGAUUGUUU; siDR5#2: ACAAUCAAGACUACG GCUGUU. To evaluate the apoptosis of RA FLSs, RA FLSs were transfected with siRNA (100 nM), Lv-IL13Rα1, or their respective controls in the presence of IL13, CoCl_2_, or Tm for 24 h, respectively. Next, the cells were trypsinized and collected for detection of cell viability apoptosis with an Annexin V-FITC apoptosis detection kit according to the manufacturer’s protocol.

### Cell viability

RA FLSs were inoculated into a 96-well plate with 3000 cells/well, and three parallel duplicate wells were set in each group. After indicated treatments, 20 μL/well MTS reaction solution was added at 24 h, 48 h, and 72 h, respectively. The culture dish was placed in an incubator with 5% CO_2_ under 37 °C to incubate 2 h. The absorbance value (OD) was tested at a wavelength of 490 nm with an automatic microplate reader, and the growth curve was traced.

### Western blot and immunoprecipitation (IP)

Cells were collected and lysed with RIPA buffer plus protease inhibitor cocktail (Roche Applied Science) [2]. For the chase assay of DR5 protein stability, CHX (100 μg/ml) was introduced for the indicated times. For IP, 10% of the lysate was set aside as the input control [2]. Cleared lysates were incubated with the indicated antibodies (1 μg) and subsequent Protein A-coupled magnetic Dynabeads (50 μl from a stock solution; Thermo Fisher Scientific). Samples were subjected to standard SDS-PAGE, and the resulting bands were transferred onto polyvinylidene fluoride membrane for visualizing specific proteins [2].

Primary antibodies used in our study are described as followings: IL13Rα1 (ab79277, Abcam), IL13Rα1 (sc101382, Santa-Cruz Biotech), Trail (27,064, Proteintech), DR5 (ab199357, Abcam), ChoP (15,204, Proteintech), STAT6 (5397, Cell Signaling), phosphor-STAT6 (Tyr641) (5397, Cell Signaling), Bax (50,599, Proteintech), and Bcl (12,789, Proteintech). We used normal rabbit and mouse IgG (Santa Cruz Biotechnology, Inc.) as IP controls. The secondary antibodies used were HRP-coupled anti-mouse IgG and anti-rabbit IgG (GE Healthcare). Antibody binding was detected by enhanced chemiluminescence with hyperfilm ECL or an RGB 600 Imager (GE Healthcare).

### Quantitative real-time polymerase chain reaction (RT-qPCR) analysis

RNA extraction and RT-qPCR analysis were performed as previously described [24]. We used the following primer sets: for human IL13Rα1, 5-CGCGCCTACGGAAACT CA-3 (forward) and 5-GGACCCCACTTGCAGACAA A-3 (reverse); human β-actin, 5-TCATTCCAAATATGAGATGCGTTGT-3 (forward) and 5-GCTATCACCTCCCCT GTGTG-3 (reverse); human trail, 5-TGCGTGCTGATCGTGATCTT-3 (forward) and 5-TCTTGGAGTCTTTCTAACGAGC (reverse). The analysis was performed using the 2^-ΔΔCT^ method.

### Immunohistochemistry (IHC) and immunofluorescence (IF)

IHC was described as previously reported [24]. Briefly, the slides were incubated overnight with an anti-IL13Rα1 antibody. For immunofluorescence, cells were sequentially probed with primary antibodies and fluorescent-labeled secondary antibodies (Jackson Immunoresearch, West Grove, PA). Images were captured under a confocal microscope (FV3000, OLYMPUS, Tokyo, Japan).

### Histopathological examination

Histopathological quantification was made by investigators in a blinded manner from images of three fields representing the distal interphalangeal joint, the proximal interphalangeal joint, and joints in the carpal region and averaged over all joints in one limb of each animal. Inflammation was scored 0 to 5 according to the following criteria: 0, normal; 1, minimal inflammatory infiltration; 2, mild infiltration; 3, moderate infiltration with moderate edema; 4, marked infiltration with marked edema; and 5, severe infiltration with edema. Hyperplasia was defined as synovial tissue intimately invading the bone and/or cartilage and was scored 0 to 3 as follows: 0, none; 1, minimal; 2, moderate; and 3, severe. The bone degradation was scored using the following 0 to 3 scale: 0, no bone erosion; 1, mild surface erosion; 2, moderate surface erosion; and 3, strong surface erosion. Sections were also stained by Safranin O/Fast green kit (Solarbio) for detection of cartilage damage (0: no changes; 1: erosion in part of the cartilage surface; 2: erosion of the cartilage surface and cartilage destruction; and 3: cartilage erosion and destruction, combined with a cartilage covered by connective tissue).

### GST pull-down assay and mass spectrometry (MS)

Bacterially expressed GST-conjugated human IL13Rα1 (GST- IL13Rα1) or control GST (both 500 μg) bound to Glutathione-Sepharose 4B beads (Amersham Pharmacia, Piscataway, NJ, USA) was incubated with equal amounts of whole-cell lysates of RA FLSs at 4 °C for overnight. The washed complexes were then eluted by boiling in SDS sample buffer, separated by SDS-PAGE, and were incubated with anti-GST-conjugated beads for 24 h in a cold room. The immunoprecipitates were identified by mass spectrometry (BGI, China).

### Collagen-induced arthritis

Chick collagen type II (CCII) (2 mg/mL; Chondrex, Inc., Redmond, WA, USA) was mixed with complete Freund’s adjuvant (CFA) (Chondrex, Inc) and injected intradermally on day 0 at the base of the tail of with 100 μL of an emulsion into 8- to 11-week-old DBA/1J mice. On day 21, mice received an intradermal booster injection with 100 μg of CCII in incomplete Freund’s adjuvant (IFA) (Chondrex, Inc). To investigate the treatment efficacy of IL13Rα1 at disease onset, Lv-mouse IL13Rα1 or its control Lv-Ctrl with GFP tag was injected into the knee joints of DAB/1J mice. The thickness of the knee joints was measured using Vernier calipers. The clinical arthritis score was calculated as described previously [24]. The maximum possible score per mouse was 12. Experiments were performed using 10 mice per group. Histological analysis of knee joints was fixed in 10% buffered formalin for 48 h and decalcified in 15% EDTA. The paws were then embedded in paraffin, and 5-μm sagittal serial sections of whole knee joints were cut. Tissue sections were stained with hematoxylin and eosin.

### Statistical analysis

The arthritis scores, paw swelling, and cell viability were analyzed with the Mann-Whitney *U* test as the requirement of Gaussian distribution and homogeneity of variance were not met. Differences in the levels of apoptosis, trail expression, apoptotic rate, and Bax/Bcl ratio between the groups were analyzed with one-way ANOVA for homogeneity test of variance, and we fulfilled nonparametric Mann-Whitney *U* test for the comparison between two groups due to the heterogeneity of variances. IL13Rα1 expression levels at both mRNA and protein levels and histological score were confirmed to Gaussian distribution and homogeneity of variances and were compared with Student’s unpaired *t* test. Data were expressed as mean ± standard deviation (SD) of the means. SPSS software (version 22.0; SPSS Inc., Chicago, IL, USA) was used to accomplish all statistical analysis. Two-sided *P* values were considered as statistical significance if less than 0.05.

## Results

### IL13Rα1 expression in the synovial tissues and fibroblasts from RA patients

**Fig. 1:**
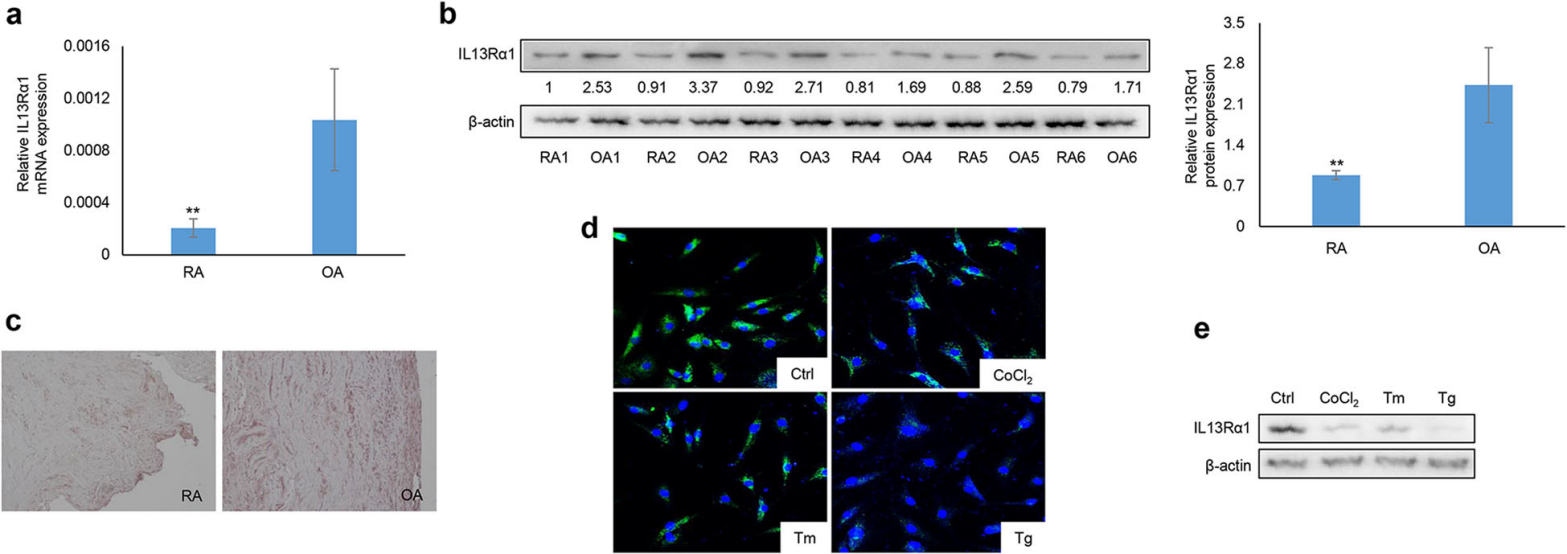
IL13Rα1 is responsive to ER stress and promotes apoptosis of RA FLSs. **a**–**c** RT-qPCR, Western blot (WB), and IHC analyses of IL13Rα1 in the synovial tissues from RA (*n* = 12) and OA (*n* = 8) patients. Band intensity given underneath gel images was measured using ImageJ software (NIH, Bethesda, MD, USA) and presented as fold change compared with the first RA sample. **d** IF analysis of IL13Rα1 (green) in RA FLSs with the stimulation of Tunicamycin (Tm, 5 μg/mL), Thapsigargin (Tg, 100 nM), or cobalt chloride (CoCl_2_, 10 μM) for 12 h. **e** IB analyses of IL13Rα1 in RA FLSs with the stimulation of Tm, Tg, and CoCl_2_ as indicated doses for 12 h. Data in **d** and **e** represent three independent experiments from three different RA samples with similar results. **p < 0.01compared with RA

To study the role of IL13Rα1 in RA, its expression in the synovial tissues was firstly analyzed. The expression of IL13Rα1 at both mRNA (Fig. 1a) and protein (Fig. 1b) levels were relatively lower in the synovial tissues from RA patients when compared to those from OA patients. Similar results of IL13Rα1 staining was also duplicated by the IHC analysis (Fig. 1c, Supplementary Fig. 1a). In addition, IF analysis demonstrated that IL13Rα1 was mainly localized in the cytoplasmic compartment of FLSs (Fig. 1d, Supplementary Fig. 1b). We also analyzed the expression of IL13Rα1 in RA FLSs with the exposure of CoCl_2_, Tm, or Tg. Of them, CoCl_2_, as an inducer to mimic hypoxic condition, could also induce ER stress [25]. The data showed that IL13Rα1 protein decreased obviously when RA FLSs were stimulated with CoCl_2_, Tm, or Tg (Fig. 1d and e). These results suggest that IL13Rα1 may be involved in the cellular response of RA FLSs towards ER stress.

### Effect of IL13Rα1 on viability and apoptosis of RA FLSs

**Fig. 2:**
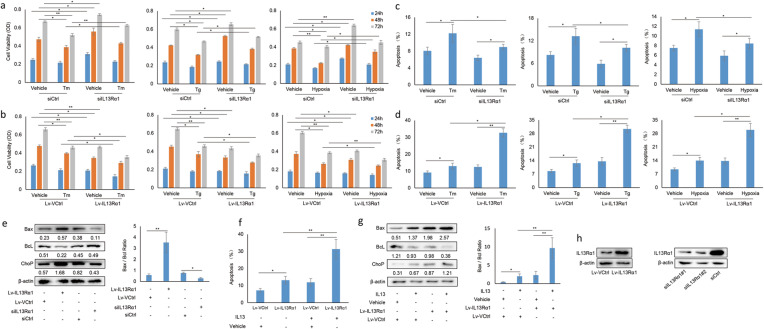
Effects of IL13Rα1 on cell viability and apoptosis of RA FLSs. **a** Following transfection with siIL13Rα1 or its negative control (siCtrl), cell viability of RA FLSs (*n* = 3) was measured by MTS post stimulation with Tg (100 nM), Tm (5 μg/mL), or CoCl_2_ (10 μM) for 12 h. **b** RA FLSs (*n* = 3) harboring the lentiviral-Myc-tagged IL13Rα1 (Lv-IL13Rα1) or its control (Lv-Ctrl) was subjected to MTS for cell viability analysis. **c**, **d** After treatment as indicated in **a** and **b**, the apoptotic rate of RA FLSs (*n* = 3) was assessed by Annexin V staining. **e** Whole lysates of RA FLSs (*n* = 3) were subjected for Western blot by anti-Bax, anti-Bcl, and anti-ChoP antibodies (left). Band intensities are quantified and the Bax/Bcl ratio is summarized into bar charts (right). **f** RA FLSs (*n* = 3) harboring Lv-IL13Rα1 or Lv-Ctrl were stimulated with IL-13, cell apoptosis was assessed by Annexin V staining. **g** Whole lysates from RA FLSs (*n* = 3) harboring Lv-IL13Rα1 or Lv-Ctrl were subjected for western blot by anti-Bax, anti-Bcl, and anti-ChoP antibodies (left). Band intensities are quantified and the Bax/Bcl ratio is summarized into bar charts (right). **h** Silencing efficiency of siIL13Rα1 was confirmed by Western blot. The two siRNAs were combined at equal concentrations for the subsequent experiments. IL13Rα1 in RA FLSs was also detected by Western blot following transfection with Lv-IL13Rα1 or Lv-Ctrl. Data are representative of three independent experiments from three different RA samples (*n* = 3) with similar results. *p < 0.05, **p < 0.01 vs the mean ± SD of siCtrl or Lv-Ctrl in the presence or absence of ER stress inducer (Tg, Tm, and CoCl_2_) or IL-13

In vitro experiments were then performed to investigate whether IL13Rα1 could affect the biological activity of RA FLSs. Firstly, the cell viability increased more rapidly when IL13Rα1 was silenced as compared to its negative control siCtrl (Fig. 2a). Moreover, the cell viability of RA FLSs decreased following the challenge of Tm, Tg, and CoCl_2_. However, such reduction could be restored by silencing IL13Rα1 (Fig. 2a). In contrast, the decrease of cell viability due to the challenge of Tm, Tg, and CoCl_2_ became more obvious when IL13Rα1 was overexpressed by lentiviral transfection (Lv-IL13Rα1) as compared with its vector control (Lv-Ctrl) (Fig. 2b).

Then, we analyzed the apoptosis of RA FLSs when IL13Rα1 expression was silenced or overexpressed. The data showed that the apoptotic proportions of RA FLSs decreased (Fig. 2c) or increased (Fig. 2d) respectively, when IL13Rα1 was silenced or overexpressed as compared to their parental control ones. Additionally, the increase of apoptotic RA FLSs due to CoCl_2_, Tm, or Tg challenge could be attenuated by silencing IL13Rα1 (Fig. 2c), but became more obvious by overexpressing it (Fig. 2d). Bax or Bcl-2 represents pro-apoptotic or anti-apoptotic markers and the Bax/Bcl-2 ratio serves to determine cell susceptibility to apoptosis [26]. Further analysis by Western blot supported that the Bax/Bcl-2 ratio decreased significantly in RA FLSs when IL13Rα1 was knocked down, but enhanced more obviously when IL13Rα1 was overexpressed (Fig. 2e).

In this study, we also analyzed the effect of IL13Rα1 on cell apoptosis in the presence of its ligand, IL-13. Although IL-13 treatment induced a marginal increase of the apoptotic rate in RA FLSs, such an increase became more obvious by overexpressing IL13Rα1 simultaneously (Fig. 2f). A similar increase of Bax/Bcl-2 ratio was also found when IL-13-stimulated RA FLSs were overexpressed by IL13Rα1 (Fig. 2g). However, no obvious increase of apoptosis was observed when IL13Rα1 was overexpressed in OA FLSs (Supplementary Fig. 2). Furthermore, the expression of IL13Rα1 in RA FLSs following transfection with siIL13Rα1/siCtrl or Lv-IL13Rα1/Lv-Ctrl were shown in Fig. 2h.

### IL13Rα1 promotes cell apoptosis of RA FLSs through upregulating trail

**Fig. 3:**
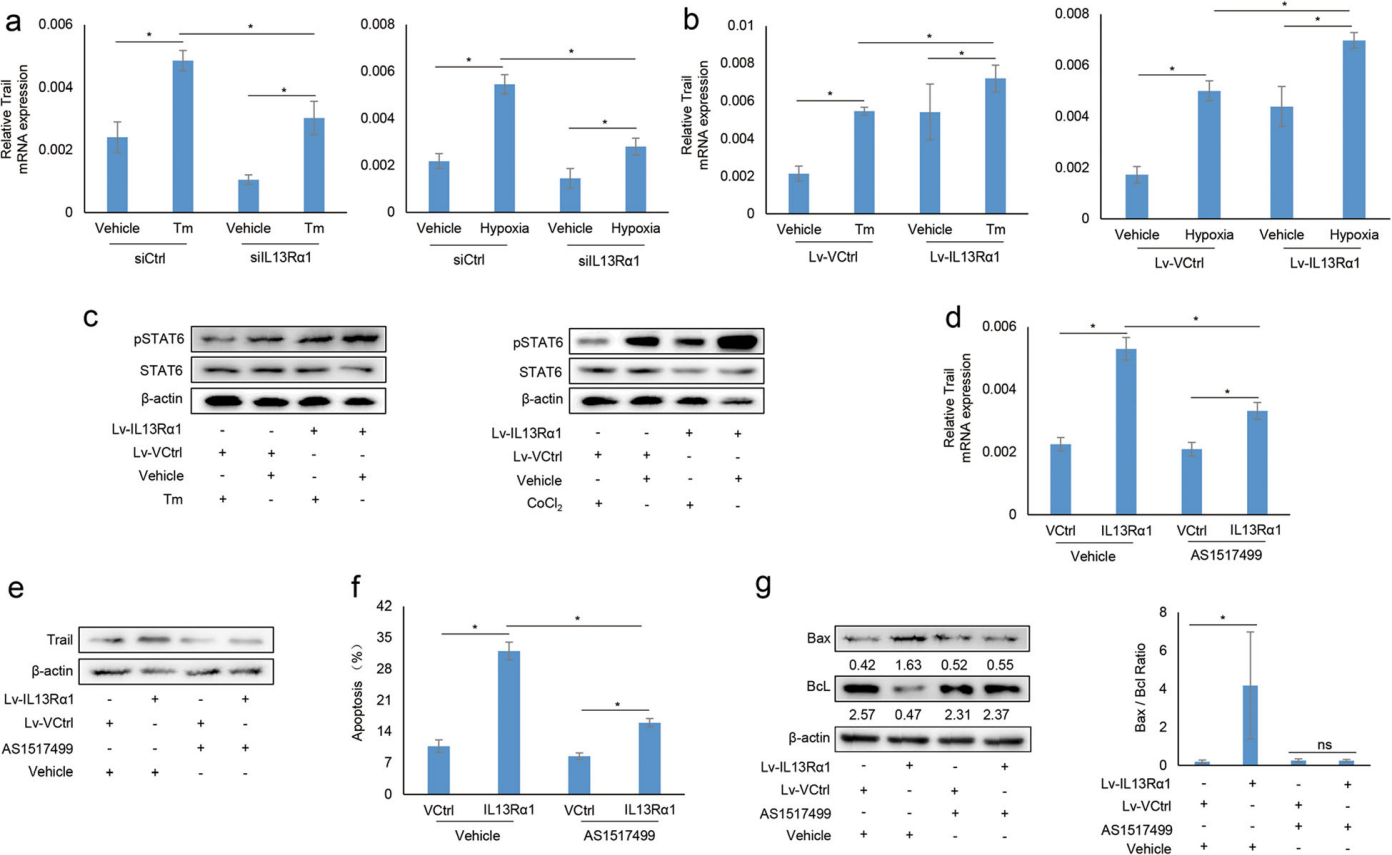
Effects of IL13Rα1 on trail expression in RA FLSs. **a** RA FLSs (*n* = 3) were transfected with siIL13Rα1 or siCtrl. Total RNA was extracted and subjected to qRT-PCR for analyzing the changes of trail expression. **b** The expression of trail at RNA level was detected when RA FLSs (*n* = 3) was transfected with Lv-IL13Rα1 or Lv-Ctrl. **c** Phosphorylated and total STAT6 protein were detected in Lv-IL13Rα1 or Lv-Ctrl transfected RA FLSs following Tm or CoCl_2_ stimulation. **d**, **e** RA FLSs (*n* = 3) harboring Lv-IL13Rα1 or Lv-Ctrl were stimulated with STAT6 inhibitor. Total RNA and protein were subjected to qRT-PCR (**d**) and Western blot (**e**) for analyzing trail expression at both RNA and protein levels. With similar treatment in **d** and **e**, cell apoptosis (**f**) and Bax or Bcl protein (**g**) were detected by Annexin V staining and Western blot. Band intensities are quantified and the Bax/Bcl ratio is summarized into bar charts (right). Data are expressed as the mean of three RA samples ± SD and represent three independent experiments (*n* = 3). Data in **a** and **b**: *p < 0.05 vs the mean ± SD of siCtrl or Lv-Ctrl in the presence of ER stressor. Data in **d**, **f**, and **g**: *p < 0.05 vs the mean ± SD of Lv-IL13Rα1 with vehicle

Trail-DR5-mediated signaling is important for the apoptosis of RA FLSs [15]. In this study, we found that the expression of the trail in RA FLSs decreased when IL13Rα1 was silenced (Fig. 3a), but increased when IL13Rα1 was overexpressed (Fig. 3b) as compared with their parental control ones. IL13Rα1 was reported that it could activate STAT6 after binding to IL13 [8]. Here, our data showed that the phosphorylated levels of STAT6, but not its total protein levels, increased in RA FLSs when IL13Rα1 was overexpressed (Fig. 3c), and such increase was still obvious when RA FLSs were stimulated by Tm or CoCl_2_ simultaneously. To determine whether IL13Rα1 regulated trail expression through STAT6, Lv-IL13Rα1-, or Lv-Ctrl-transfected FLSs was treated with STAT6 inhibitor, AS1517499. The data showed that AS1517499 treatment could attenuate the increase of the trail resulting from IL13Rα1 overexpression (Fig. 3d and e). Furthermore, the increase of apoptotic rate (Fig. 3f) and the Bax/BcL ratio (Fig. 3g) resulting from IL13Rα1 overexpression could be reverted when RA FLSs were treated with AS1517499. These results suggest that IL13Rα1 induces the expression of trail via STAT6.

### IL13Rα1 interacts and stabilizes DR5 protein in RA FLSs

**Fig. 4:**
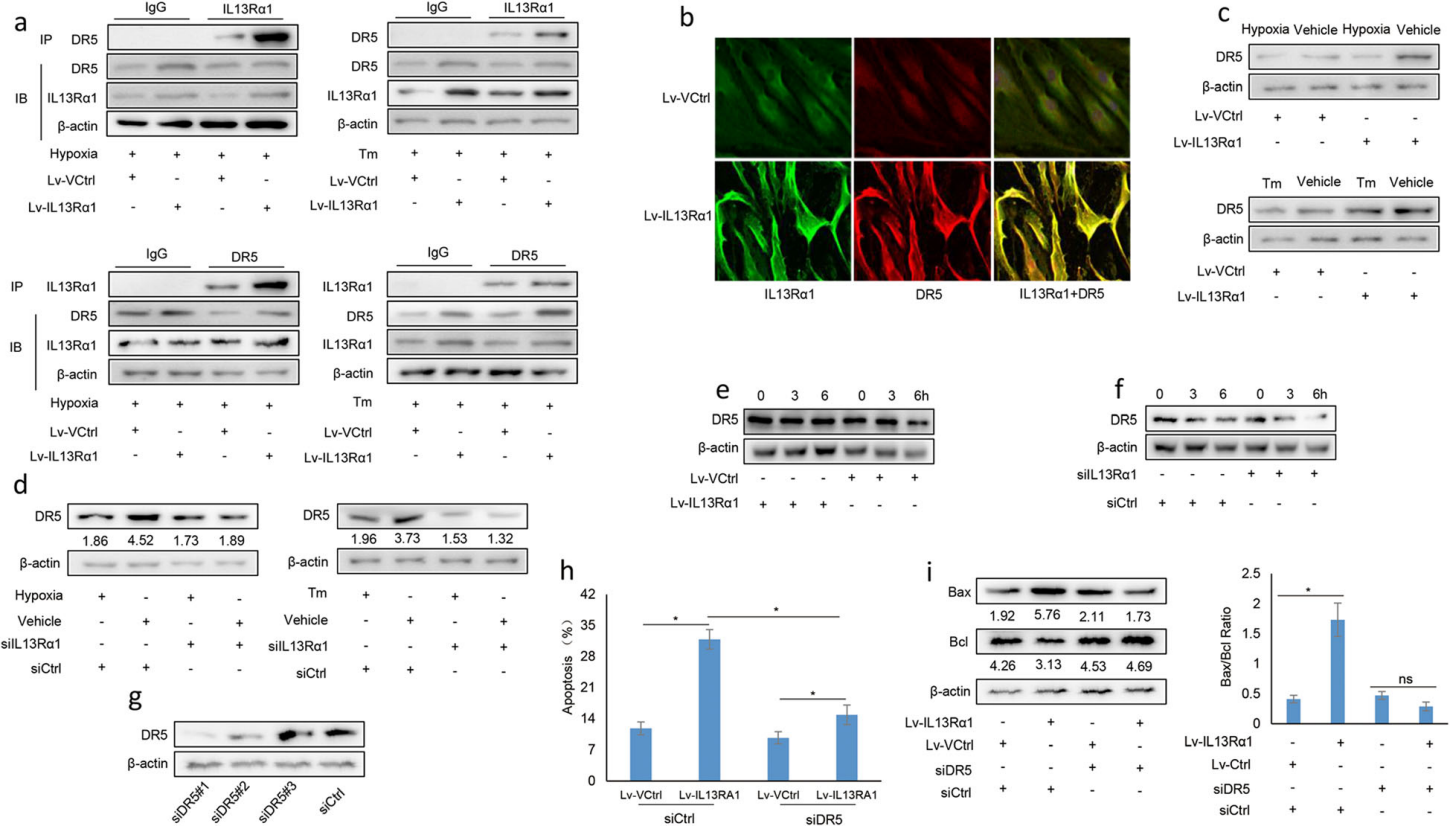
IL13Rα1 interacts and stabilizes DR5. **a** Whole lysates from CoCl_2_ or Tm challenged RA FLSs were IP with anti-IL13Rα1 or anti-DR5 and IB by anti-DR5 or anti-IL13Rα1. **b** After transfection with Lv-IL13Rα1 or Lv-Ctrl for 24 h, IL13Rα1 (green) and DR5 (red) in RA FLSs from three samples were detected by IF. **c** Western blot of DR5 protein in RA FLSs following transfection with Lv-IL13Rα1 relative to Lv-Ctrl. **d** With the exposure of CoCl_2_ or Tm, RA FLSs were transfected with siIL13Rα1/siCtrl for 24 h, DR5 protein was measured by Western blot, and band intensity given underneath gel images was measured using ImageJ software. **e**, **f** With the exposure of CHX plus Tm, total proteins from Lv-IL13Rα1/Lv-Ctrl-transfected or siIL13Rα1/siCtrl-transfected RA FLSs were subjected to Western blot for analyzing DR5 expression as indicated time points. Band intensity was measured using ImageJ software and presented as fold change compared as indicated. **g** Silencing efficiency of siRNA targeting DR5 was detected by Western blot, and these two siRNAs were combined at an equal concentration for the following experiments. RA FLSs (*n* = 3) harboring Lv-IL13Rα1 or Lv-Ctrl were transfected with siDR5 or siCtrl, and the apoptotic rate (**h**) and Bax or Bcl protein (**i**) were detected by Annexin V staining and western blot. Band intensities are quantified and the Bax/Bcl ratio is summarized into bar charts (right). Data are representative of three independent experiments from three different RA samples (*n* = 3) with similar results. ns, not significant vs the mean ± SD of Lv-Ctrl in the presence of siDR5; *p < 0.05 vs the mean ± SD of IL13Rα1 in the presence of siCtrl

To further elucidate the mechanism of IL13Rα1 in RA, GST pull-down plus mass spectrum was applied. Among its potential interactors (Supplementary Table 2), DR5 was selected for further analysis as it is closely related to cell apoptosis. Our data showed that IL13Rα1 and DR5 reciprocally interacted in RA FLSs and their co-precipitation efficacy increased when RA FLSs were introduced by IL13Rα1 in the presence of CoCl_2_ or Tm (Fig. 4a). Co-localization of IL13Rα and DR5 was also confirmed by IF analysis (Fig. 4b). Furthermore, with the stimulation of CoCl_2_ or Tm, IL13Rα1 overexpression upregulated (Fig. 4c), while silencing IL13Rα1downregulated the expression levels of DR5 protein in RA FLSs (Fig. 4d). Detailed analysis showed that DR5 protein degraded more slowly in IL13Rα1-overexpressing RA FLSs when compared with its control (Fig. 4e). However, with the stimulation of CoCl_2_, DR5 protein degraded more rapidly in siIL13Rα1-transfected RA FLSs when compared with siCtrl-transfected ones (Fig. 4f). To further elucidate the involvement of DR5 in the pro-apoptotic effect of IL13Rα1, siRNA targeting DR5 was applied in RA FLSs with IL13Rα1 overexpression, and the silencing efficiency of DR5 was confirmed by Western blot (Fig. 4g). We found that the induction of apoptosis resulting from IL13Rα1 overexpression, as evidenced by the increase of Annexin V staining and Bax/Bcl-2 rate, could be attenuated by silencing DR5 in RA FLSs (Fig. 4h and i). Thus, DR5 was also involved in the pro-apoptotic effect of IL13Rα1.

### IL13Rα1 ameliorate the arthritis phenotype of RA

**Fig. 5:**
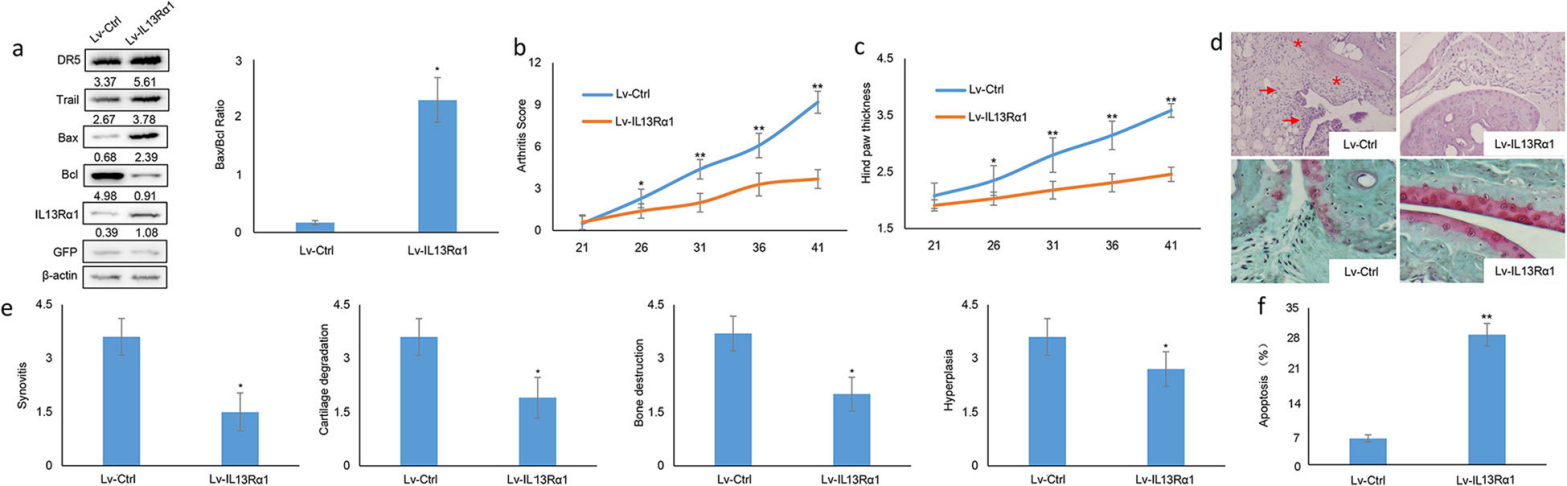
Treatment with IL13Rα1 halts and reverses the development of CIA. **a** The synovial tissues from knee joints in Lv-IL13Rα1 or Lv-Ctrl transfected CIA mice (*n* = 10 per group) at the resolution phase of arthritis were homogenized in cell lysis buffer for Western blot detection of IL13Rα1, DR5, Trail, Bax, Bcl, and GFP (left). Band intensities are quantified and the Bax/Bcl ratio is summarized into bar charts (right). **b**, **c** Comparison of the clinical score (**b**) and paw swelling (**c**) between arthritic mice treated with Lv-Myc-IL13Rα1 (*n* = 10) or Lv-Ctrl (*n* = 10). **d** Representative images of hematoxylin-eosin (H&E, upper) and Safranin O/Fast green staining (lower) of the knee joint. The synovial thickening (red arrow) and bone erosion (red asterisk) are shown (*n* = 10). **e** Inflammation, hyperplasia, cartilage degradation, and bone destruction as evaluated through the scoring system (*n* = 10 per group). **f** FLSs were isolated from knee joints of CIA models (*n* = 6 per group) and subjected to flow cytometry for analyzing apoptosis. *p < 0.001 vs the mean ± SD of indicated parameters in Lv-Ctrl

To assess the therapeutic potential of IL13Rα1, CIA models were treated with Lv-IL13Rα1 and Lv-Ctrl (10^9^ PFU per joint) and the study period extended up to 21 days post injection. The presence of GFP in the synovial tissues as evidenced by Western blot analysis confirmed the transfection efficiency of Lv-IL13Rα1 and Lv-Ctrl (Fig. 5a). The results show that the articular index (Fig. 5b) and knee joint thickness (Fig. 5c) were obviously ameliorated post-intra-articular injection of Lv-IL13Rα1 when compared with its controls. Histological analysis of the joints revealed less degree of synovial hyperplasia, inflammation, cartilage destruction, and bone erosion with Lv-IL13Rα1 injection (Fig. 5d and e). To confirm such therapeutic effect of Lv-IL13Rα1 injection was achieved by direct targeting FLSs, our data confirmed that, besides the increase of IL13Rα1, DR5, and Trail, Bax/Bcl ratio increased significantly as well in synovial tissues from Lv-IL13Rα1 injected ones (Fig. 5a). FLSs were also freshly isolated from the CIA models and the apoptotic rate increased significantly in FLSs when introducing Lv-IL13Rα1 in contrast to Lv-Ctrl (Fig. 5f). Collectively, these data support that IL13Rα1 plays an anti-arthritis role in RA.

## Discussion

Previous study demonstrated that ER stress-associated gene signatures are highly expressed in RA synovium [6]. In addition, GRP78 and Synoviolin could prevent RA FLSs against ER stress-induced apoptosis and contribute to the chronic inflammation and joint destruction [3, 6, 27]. Thus, identifying key factors accounting for the apoptotic resistance of RA FLSs against ER stress would hold great promise for RA intervention. Previous study demonstrated that IL13Rα1 regulates the cellular responses to various stressful stimulations that it could promote more fibrosis in response to transforming growth factor-β (TGF-β) [28]. Likewise, neuronal IL13Rα1 increases the susceptibility to oxidative damage [29]. In this study, our loss- and gain- of functional experiments argued IL13Rα1-mediated signaling pathway as a novel molecular program to counter the apoptotic resistance of RA FLSs towards ER stress. Integrating the data obtained from the in vitro studies about ER stress-challenged RA FLSs and the in vivo experiments that transduced IL13Rα1 into CIA models enabled us to deduce that targeting IL13Rα1 may provide novel treatment strategy for RA. In addition, the relatively lower levels of IL13Rα1 expression at steady state in RA FLSs and the further decrease in response to ER stress may explain the reason why RA FLSs are apoptosis resistance against ER stress.

Although the exact roles of IL-13 in RA were paradoxical from previous studies [9–11], in this study, we confirmed the anti-inflammatory role of IL13 in RA that it requires IL13Rα1 to induce apoptosis of RA FLSs. Additionally, we also defined the molecular mechanism underlying how IL13Rα1 combats the apoptotic resistance of RA FLSs against ER stress that IL13Rα1 could activate Trail-DR5 signaling pathway through upregulating the expression of the trail via activating STAT6 and simultaneously interact and stabilize the receptor of the trail, DR5. More importantly, IL13Rα1 overexpression could promote cell apoptosis even in the absence of its ligand IL13 and ER stress inducer. These facts indicate that IL13Rα1 acts as a scaffold in the membrane to activate DR5 and STAT6, respectively, and the change of IL13Rα1 level (quantity) is more important other than its ligands to regulate cell apoptosis.

As a TNF superfamily member, trail protects against the inflammation progression of RA [30]. Furthermore, the local administration of trail inhibits the development of arthritis, whereas the blockade of trail-mediated apoptosis increases the occurrence and severity of arthritis in CIA models [31]. However, the application of the trail as a therapeutic agent might be questioned because of the ability of the soluble trail to induce apoptosis of normal hepatocytes [32]. Here, our data showed that the pro-apoptotic effects of IL13Rα1 on OA FLSs are not as obvious as those on RA FLSs, indicating the disease-specific role of IL13Rα1 in RA. Although our in vivo experiments showed that IL13Rα1 could be transferred into synovial tissues by lentiviral vector and thus conferred synovial tissues with a pro-apoptotic feature, we could not exclude the possibility that T cells, B cells, and monocytes/macrophages, which are involved in immune-mediated inflammation, might also be targets for IL13Rα1-mediated apoptosis.

## Conclusion

Together, our findings provide an alternative option to overcome apoptosis resistance to ER stress of RA FLSs and a novel treatment strategy for RA.

## Supplementary information

**Additional file 1: Fig. S1.** Negative control for IHC and IF analysis. (**a**) Synovial tissues from RA and OA patients were subjected to IHC analysis with primary antibody against IgG instead of IL13Rα1. (**b**) RA FLSs with the stimulation of CoCl_2_, Tm and Tg were subjected to IF analysis with primary antibody against IgG instead of IL13Rα1. (**c**) RA FLSs transfected with Lv-Myc-IL13Rα1 or Lv-Ctrl were subjected to co-focal immunofluorescent analysis with primary antibody against IgG instead of IL13Rα1 or DR5.

**Additional file 2: Fig. S2.** Effects of IL13Rα1 on apoptosis of OA FLSs. FLSs were isolated from OA (*n* = 3 per group) and subjected to flow cytometry for analyzing apoptosis. ns, not significant vs the mean ± SD of indicated parameters in Lv-Ctrl.

**Additional file 3: Table S1** Clinical characteristics of RA patients.

**Additional file 4: Table S2** List of potential IL13Rα1 partners in RA FLSs identified by mass spectrometry.

## Data Availability

The datasets used and analyzed during the current study are available from the corresponding author on reasonable request.

## Abbreviations

RA: Rheumatoid arthritis
FLSs: Fibroblast-like synoviocytes
ER: Endoplasmic reticulum
UPR: Unfolded protein response
Tg: Thapsigargin
Tm: Tunicamycin
siRNA: Small interfering RNA
IP: Immunoprecipitation
RT-qPCR: Quantitative real-time polymerase chain reaction
IHC: Immunohistochemistry
IF: Immunofluorescence
MS: Mass spectrometry
CCII: Collagen type II
CFA: Complete Freund’s adjuvant
IFA: Incomplete Freund’s adjuvant
TGF-β: Transforming growth factor-β
STAT6: Transducer and activator of transcription 6
CIA: Collagen-induced arthritis
CoCl_2_: Cobalt chloride 2
DMEM: Dulbecco’s modified Eagle’s medium
