# Correction: IL13Rα1 protects against rheumatoid arthritis by combating the apoptotic resistance of fibroblast-like synoviocytes

**DOI:** 10.1186/s13075-025-03540-9

**Published:** 2025-03-25

**Authors:** Xiaomei Yang, Qingwei Guo, Tingting Feng, Qiqi Lu, Luna Ge, Jihong Pan, Kehong Bi, Li Qiao, Lei Tian, Tianhua Xie, Chengfang Yao, Guanhua Song, Lin Wang

**Affiliations:** 1 Department of Hematology, Qilu Children’s Hospital of Shandong University, Jinan, China; 2 Shandong Provincial Qianfoshan Hospital, Shandong University, Jinan, China; 3 Department of Pathology, Shandong University Medical School, Jinan, China; 4 Department of Rheumatology and Autoimmunology, The First Affiliated Hospital of Shandong First Medical University, Key Lab for Biotech-Drugs of National Health Commission, Key Lab for Rare & Uncommon Diseases of Shandong Province, Shandong Medicinal Biotechnology Centre, Jinan, 250062, China; 5 School of Medicine and Life Sciences, University of Jinan-Shandong Academy of Medical Sciences, Jinan, China; 6 Department of Joint Surgery, Shandong Provincial Hospital Affiliated to Shandong University, Jinan, China; 7 Department of Rheumatology, Shandong Provincial Hospital Affiliated to Shandong University, Jinan, China; 8 Institute of Basic Medicine, Shandong First Medical University & Shandong Academy of Medical Sciences, 250062 Jinan, China

## **Correction: Arthritis Res Ther 22, 184 (2020)**

10.1186/s13075-020-02270-4.

Following publication of the original article [1], the authors reported an error in Fig. 5D. The images of haematoxylin-eosin (H&E) and Safranin O/Fast green staining in Fig. 5d was mistakenly used, as more than one pathological gene, besides IL13Rα1 was investigated. The corrected Fig. 5D is provided below.

**Fig. 5:**
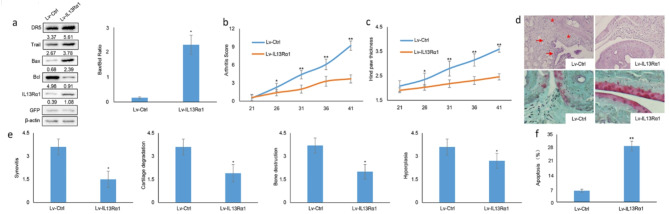
Incorrect Figure

**Fig. 5:**
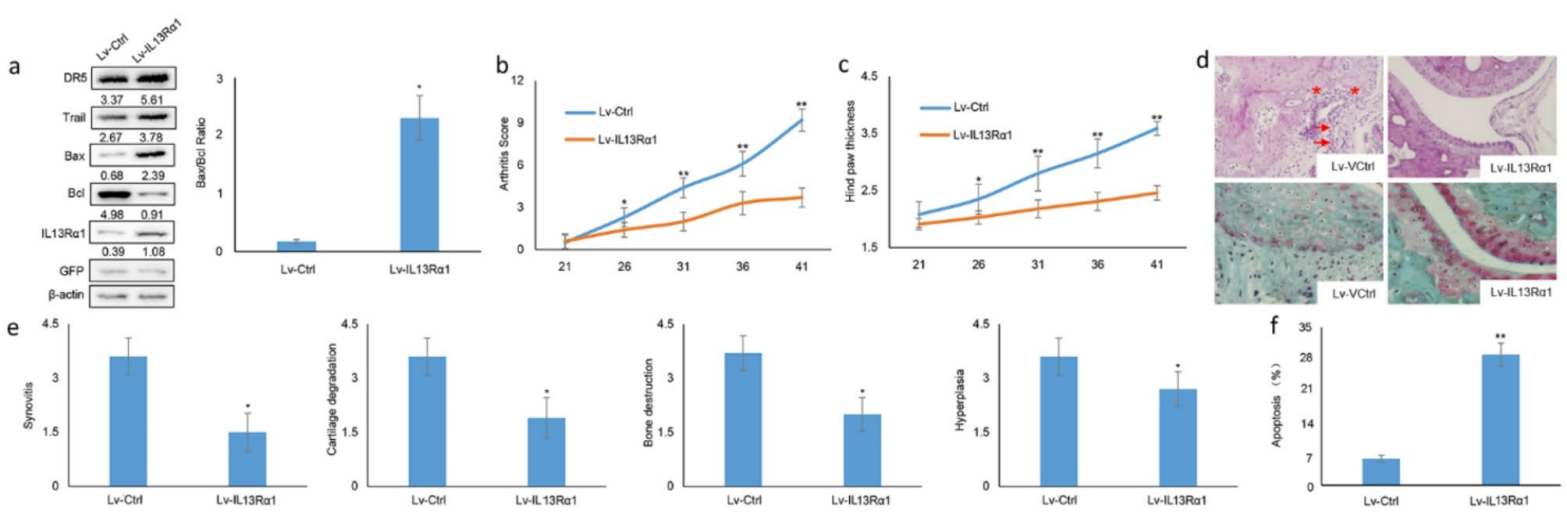
Correct Figure
