# Supplementary figures and images for: IL13Rα1 protects against rheumatoid arthritis by combating the apoptotic resistance of fibroblast-like synoviocytes
Source: Arthritis Res Ther. 2020 Aug 8;22:184. doi: 10.1186/s13075-020-02270-4 (PMC7414989; doi:10.1186/s13075-020-02270-4)

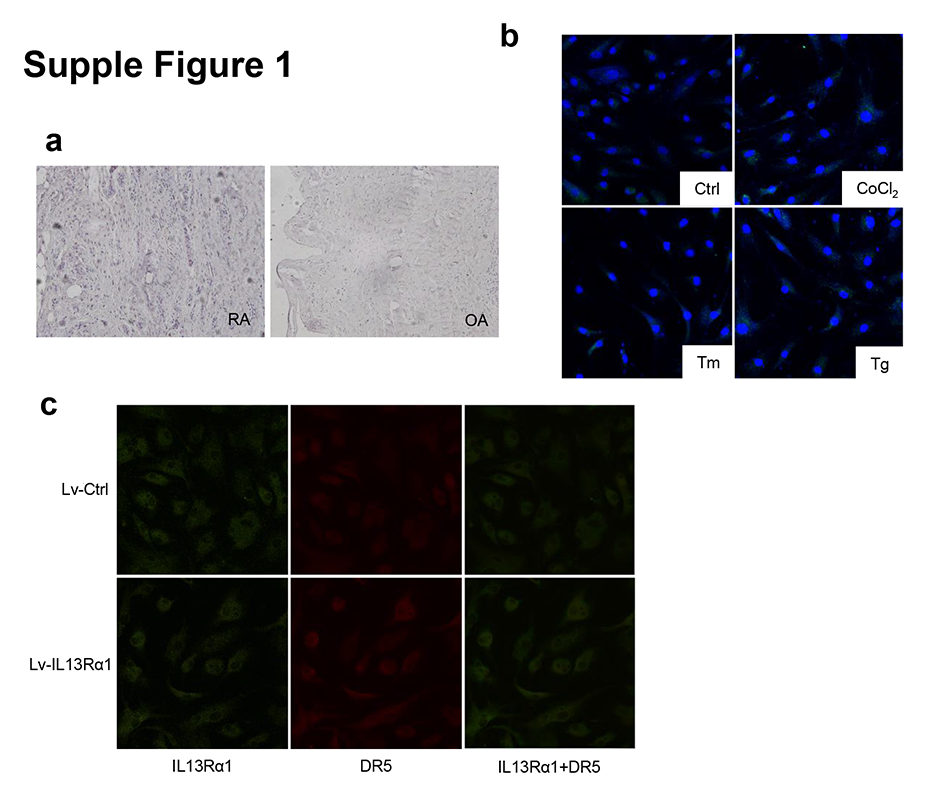

Supplement: Supplementary file 1 — Additional file 1: Fig. S1. Negative control for IHC and IF analysis. (a) Synovial tissues from RA and OA patients were subjected to IHC analysis with primary antibody against IgG instead of IL13Rα1. (b) RA FLSs with the stimulation of CoCl2, Tm and Tg were subjected to IF analysis with primary antibody against IgG instead of IL13Rα1. (c) RA FLSs transfected with Lv-Myc-IL13Rα1 or Lv-Ctrl were subjected to co-focal immunofluorescent analysis with primary antibody against IgG instead of IL13Rα1 or DR5. [file 13075_2020_2270_MOESM1_ESM.tif]

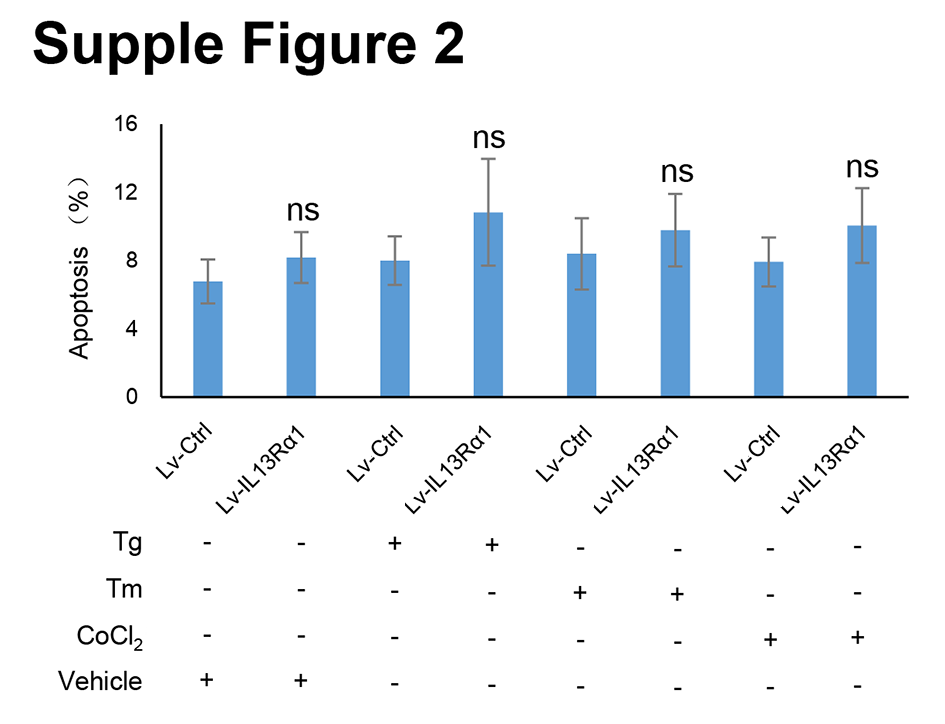

Supplement: Supplementary file 2 — Additional file 2: Fig. S2. Effects of IL13Rα1 on apoptosis of OA FLSs. FLSs were isolated from OA (n = 3 per group) and subjected to flow cytometry for analyzing apoptosis. ns, not significant vs the mean ± SD of indicated parameters in Lv-Ctrl. [file 13075_2020_2270_MOESM2_ESM.tif]
