# Supplementary material for: IL13Rα1 protects against rheumatoid arthritis by combating the apoptotic resistance of fibroblast-like synoviocytes
Source: Arthritis Res Ther. 2020 Aug 8;22:184. doi: 10.1186/s13075-020-02270-4 (PMC7414989; doi:10.1186/s13075-020-02270-4)
Supplement: Supplementary file 3 — Additional file 3: Table S1 Clinical characteristics of RA patients. [file 13075_2020_2270_MOESM3_ESM.docx]

| Supplementary Table 1. **Clinical characteristics of RA patients.** | | | | | | | | |
| --- | --- | --- | --- | --- | --- | --- | --- | --- |
| **Sample no.** | **Gender** | **Age** | **DAS28** | **RF (IE/ml）** | **CRP(mg/L)** | **Anti-CCP (E/mL)** | **Duration (mon)** |  |
| 1 | F | 56 | 3.7 | 56 | 59 | 26 | 16 |  |
| 2 | F | 65 | 4.5 | 73 | 62 | 76 | 67 |  |
| 3 | F | 34 | 5.2 | 151 | 37 | 163 | 38 |  |
| 4 | F | 49 | 4.9 | 37 | 52 | 1346 | 159 |  |
| 5 | F | 38 | 4.3 | 71 | 29 | 795 | 27 |  |
| 6 | F | 49 | 5.1 | 167 | 33 | 2685 | 66 |  |
| 7 | F | 55 | 3.8 | 55 | 71 | 331 | 79 |  |
| 8 | F | 61 | 2.9 | 8 | 59 | 92 | 95 |  |
| 9 | F | 57 | 3.7 | 12 | 69 | 75 | 51 |  |
| 10 | F | 55 | 4.9 | 86 | 34 | 1756 | 9 |  |
| 11 | F | 62 | 3.5 | 16 | 51 | 216 | 88 |  |
| 12 | F | 53 | 4.8 | 27 | 76 | 338 | 91 |  |
| 13 | F | 70 | 2.8 | 37 | 169 | 596 | 126 |  |
| 14 | F | 66 | 3.9 | 49 | 175 | 87 | 104 |  |
| 15 | M | 63 | 5.2 | 37 | 117 | 105 | 139 |  |
| 16 | M | 53 | 5.7 | 28 | 75 | 572 | 27 |  |
| 17 | M | 42 | 4.5 | 167 | 37 | 769 | 61 |  |
| 18 | M | 61 | 4.1 | 59 | 65 | 1465 | 7 |  |
| 19 | M | 55 | 4.4 | 86 | 19 | 76 | 58 |  |
| 20 | M | 43 | 5.1 | 77 | 57 | 335 | 42 |  |
| 21 | M | 37 | 4.7 | 196 | 34 | 24 | 93 |  |
| 22 | M | 50 | 4.9 | 237 | 46 | 2675 | 37 |  |
| 23 | M | 53 | 5.8 | 261 | 195 | 625 | 59 |  |
| 24 | M | 65 | 5.1 | 27 | 37 | 225 | 34 |  |
| 25 | M | 72 | 5.4 | 79 | 57 | 351 | 29 |  |
| 26 | M | 62 | 4.6 | 69 | 62 | 169 | 16 |  |
