# Supplementary material for: IL13Rα1 protects against rheumatoid arthritis by combating the apoptotic resistance of fibroblast-like synoviocytes
Source: Arthritis Res Ther. 2020 Aug 8;22:184. doi: 10.1186/s13075-020-02270-4 (PMC7414989; doi:10.1186/s13075-020-02270-4)
Supplement: Supplementary file 4 — Additional file 4: Table S2 List of potential IL13Rα1 partners in RA FLSs identified by mass spectrometry. [file 13075_2020_2270_MOESM4_ESM.doc]

| **Supplementary Table 2** List of potential IL13Rα1 partners in RA FLSs identified by mass spectrometry | | | |
| --- | --- | --- | --- |
| **Human gene name** | **Name** | **Mass score** | **Peptide number** |
| **Metabolism** | | | |
| PKM | Pyruvate kinase | 165 | 9 |
| GPI | Glucose-6-phosphate isomerase | 326 | 7 |
|  |  |  |  |
| **mRNA** |  | 227 | 8 |
| ISY1 | Pre-mRNA-splicing factor ISY1 | 329 | 10 |
| FIP1L1 | Pre-mRNA 3~-end-processing factor FIP1 | 266 | 7 |
| PRPF19 | Pre-mRNA-processing factor 19 | 169 | 7 |
|  |  |  |  |
| **Signaling pathway** | | | |
| MAP3K5 | Mitogen-activated protein kinase kinase kinase 5 | 246 | 8 |
| MAP4K4 | Isoform 4 of Mitogen-activated protein kinase kinase kinase kinase 4 | 225 | 10 |
| NEK2 | Serine/threonine-protein kinase Nek2 | 152 | 4 |
| ILK | Integrin-linked protein kinase | 179 | 7 |
| MAP3K15 | Mitogen-activated protein kinase kinase kinase 15 | 209 | 5 |
|  |  |  |  |
| **Protein processing and modification** | | | |
| HSP90AA1 | Heat shock protein HSP 90-alpha | 221 | 7 |
| WDR87 | WD repeat-containing protein 87 | 279 | 9 |
| TXNDC5 | Thioredoxin domain-containing protein 5 | 153 | 6 |
| PDIA6 | Protein disulfide-isomerase A6 | 208 | 9 |
| HSPA1A | Heat shock 70 kDa protein 1A/1B | 136 | 5 |
| HSPA6 | Heat shock 70 kDa protein 6 | 269 | 4 |
| SHPRH | E3 ubiquitin-protein ligase SHPRH | 165 | 7 |
| RNF213 | E3 ubiquitin-protein ligase RNF213 | 221 | 8 |
| FKBP4 | Peptidyl-prolyl cis-trans isomerase FKBP4 | 237 | 5 |
|  |  |  |  |
| **Cell cycle** | | | |
| CDK10 | Cyclin-dependent kinase 10 | 166 | 6 |
| LYAR | Cell growth-regulating nucleolar protein | 112 | 6 |
| CDC5L | Cell division cycle 5-like protein | 157 | 7 |
| CDK5 | Cyclin-dependent-like kinase 5 | 295 | 8 |
| DR5 | TNF Receptor Superfamily Member 10b | 227 | 7 |
|  |  |  |  |
| **Translation** | | | |
| EIF4G3 | Eukaryotic translation initiation factor 4 gamma 3 | 299 | 6 |
| RPL8 | 60S ribosomal protein L8 | 235 | 7 |
| GCN1L1 | Translational activator GCN1 | 309 | 9 |
| RRP1 | Ribosomal RNA processing protein 1 | 267 | 6 |
|  |  |  |  |
| **Matrix model** |  |  |  |
| MXRA5 | Matrix-remodeling-associated protein 5 | 253 | 6 |
| MMP14 | Matrix metalloproteinase-14 | 258 | 7 |
| MXRA7 | Matrix-remodeling-associated protein 7 | 213 | 5 |
| MMP24 | Matrix metalloproteinase-24 | 168 | 6 |
